# Non-Lethal Detection of *Ranavirus* in Fish

**DOI:** 10.3390/v15020471

**Published:** 2023-02-08

**Authors:** Catarina D. Coutinho, Charlotte E. Ford, Joseph D. Trafford, Ana Duarte, Rui Rebelo, Gonçalo M. Rosa

**Affiliations:** 1 Centre for Ecology, Evolution and Environmental Changes (cE3c), Faculdade de Ciências da Universidade de Lisboa, 1749-016 Lisboa, Portugal; 2 Zoological Society of London, Institute of Zoology, Nuffield Building, Outer Circle, London NW8 7LS, UK; 3 School of Biological and Behavioural Sciences, Queen Mary University of London, Mile End Road, London E1 4NS, UK; 4 Department of Genetics, Evolution and Environment, University College London, Gower Street, London WC1E 6BT, UK; 5 Faculdade de Medicina Veterinária, Universidade de Lisboa, 1300-477 Lisboa, Portugal; 6 Instituto Nacional de Investigação Agrária e Veterinária, I.P. (INIAV), 2780-157 Oeiras, Portugal

**Keywords:** 3Rs, invasive sampling, pathogen, prevalence, viral load

## Abstract

Emergent infectious diseases have an increasing impact on both farmed animals and wildlife. The ability to screen for pathogens is critical for understanding host–pathogen dynamics and informing better management. *Ranavirus* is a pathogen of concern, associated with disease outbreaks worldwide, affecting a broad range of fish, amphibian, and reptile hosts, but research has been limited. The traditional screening of internal tissues, such as the liver, has been regarded as the most effective for detecting and quantifying *Ranavirus*. However, such methodology imposes several limitations from ethical and conservation standpoints. Non-lethal sampling methods of viral detection were explored by comparing the efficacy of both buccal swabbing and fin clipping. The study was conducted on two Iberian, threatened freshwater fish (*Iberochondrostoma lusitanicum* and *Cobitis paludica*), and all samples were screened using qPCR. While for *C. paludica* both methods were reliable in detecting *Ranavirus*, on *I. lusitanicum*, there was a significantly higher detection rate in buccal swabs than in fin tissue. This study, therefore, reports that fin clipping may yield false *Ranavirus* negatives when in small-bodied freshwater fish. Overall, buccal swabbing is found to be good as an alternative to more invasive procedures, which is of extreme relevance, particularly when dealing with a threatened species.

## 1. Introduction

The rise of emerging infectious diseases in humans and wildlife demands sensitive and reliable screening methods for accurate diagnosis and monitoring of populations. Yet, these often require invasive sampling to obtain tissue for molecular diagnostics. Invasive techniques are known to induce both physiological and behavioural changes in the organisms [1,2,3]. Ideally, sampling methods should be able to successfully screen for pathogens, both quickly and efficiently, whilst not impacting animal welfare.

The ethics of pathogen screening is even more challenging when the target species are threatened; in these cases, lethal approaches become non-viable, calling for a refinement of the methods. Refinement is one of the key principles of the 3Rs (replacement, reduction, and refinement), aiming to improve animal welfare in research by minimising pain, suffering or distress [4]. The increasing research focus on wildlife disease highlights the need for continuous refinement approaches, especially for non-model organisms.

Ranavirosis is a widespread disease caused by a group of viruses belonging to the genus *Ranavirus,* currently considered a notifiable disease by the World Organisation for Animal Health [5]. These viruses can infect a broad range of ectothermic vertebrates and have been linked to amphibian population collapses [6,7,8], but also to episodes of mass mortality in reptiles and fish [9,10,11,12,13,14]; the latter, with particular impact in aquaculture and fish farms [15,16,17].

Surveillance, monitoring and management of *Ranavirus* epizootics rely on a number of sampling techniques for DNA collection. The use of internal organs, namely the liver, was proved to be more effective for the detection of pathogen DNA [18,19,20,21]. However, obtaining these samples is extremely invasive, as it can only occur following euthanasia or in animals already deceased. Even though *Ranavirus* can be detected in external tissues [22,23], viral detection is expected to be comparatively lower than using internal tissues [24,25].

Previous studies have shown that sampling external tissues, such as fin clips in fish, although non-lethal, can result in high levels of stress and induce drastic behavioural changes [26,27]. Whilst obviously less impactful than sampling internal organs, fin clipping is still relatively invasive and may not be appropriate in all situations. Alternatively, swab sampling has been explored as a minimally invasive method to obtain DNA from fish, and appears to bear reduced physiological and behavioural consequences when compared to fin clipping [27]. Swab samples from buccal and skin mucus have been previously found to provide host DNA quantity and quality similar to that obtained from fin tissue [28,29,30]. However, limited advances have been made toward non-lethal pathogen detection in fish, particularly in small-sized ones. The successful detection and isolation of fish viruses obtained from the swabbing of epithelial cells [31,32], as well as the consistent results obtained in amphibians [33], suggests that this approach may be a reliable method for molecular detection of viral DNA [34], and an alternative for sampling a threatened species.

The present study compares two alternative methods for *Ranavirus* detection in two species of threatened, small-bodied, freshwater fish. In particular, it tests if buccal swabs can offer a less invasive (refined) sampling method than fin clips, while retrieving a similar estimate of the viral load.

## 2. Materials and Methods

The study species are two Iberian endemic freshwater fish: the arched-mouth nase, *Iberochondrostoma lusitanicum* (Collares-Pereira, 1980), a Critically Endangered fish from SW Portugal [35] with a maximum length of about 15 cm [36]; and the southern Iberian spined loach, *Cobitis paludica* (de Buen, 1930), a Vulnerable species distributed in southern and Eastern Iberia [37], reaching up to 13 cm [38]. Sampling occurred during the summer of 2018, in the streams of Barcarena and Jamor (Oeiras municipality, Portugal), where both species are present and *Ranavirus* infection has been previously confirmed (Coutinho & Rosa, unpublished).

**Figure 1:**
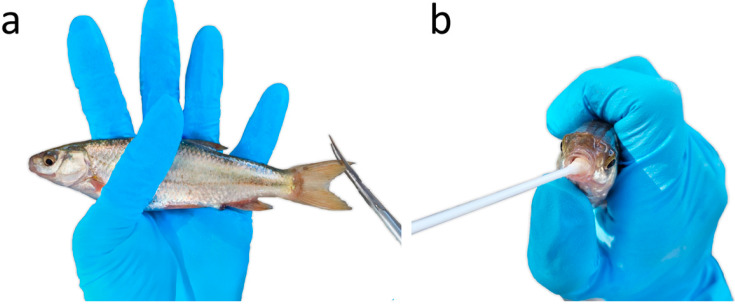
Sampling individual *Iberochondrostoma lusitanicum* fish for: (**a**) caudal fin tissue and (**b**) buccal swabs.

Animals were captured using electrofishing (SAMUS-725MP), with a frequency of 30 Hz, assisted by a dipnet. Animals were sampled while immobilised (electronarcosis), avoiding severe muscle tetany which can result in spinal injuries. A clip of 2 mm of tissue was taken from the upper portion of the caudal fin (Figure 1a) of each individual and preserved in 96% ethanol. Additionally, a mouth swab (MW113 dry swab; MWE Medical Wire, UK) was also collected: while holding the fish, we gently rubbed the swab inside the mouth, rotating it for five seconds (Figure 1b); swabs were stored dry at −20 °C. Due to the small size of the individual fish, sampling methods were followed with care not to damage gills or gill arches. Fish were released at the place of capture immediately after sampling; electroanesthesia results in fast recovery times, with no need for a withdrawal period [39].

To prevent cross-contamination between individual fish, after capture and while waiting to be processed, they were separated into different containers. Additionally, all tools were sterilised with 96% alcohol and flamed between samples, and disposable vinyl gloves were used to handle the animals [40].

DNA extraction was performed using the DNeasy Blood & Tissue Kit (Qiagen), following the manufacturer’s protocol. For buccal swabs, the tip of the swab containing the material was removed using a scalpel and placed in individual 1.5 mL Eppendorf tubes. To maximise DNA yield, the Qiagen protocol for low DNA yield, involving an initial elution of DNA using buffer AE (100 µL), was applied, which was then repeated in the same spin-column to bring the final elution volume to 200 µL. All DNA samples were stored at −20 °C.

Real-time polymerase chain reactions (qPCR) were performed following Leung et al. [41] for both *Ranavirus* DNA detection and quantification, using primers targeting a region of the major capsid protein (MCP). Samples were run in duplicate and considered positive when both wells amplified. Any disagreement between wells led to re-runs until a consensus result was achieved. To enable the quantification of viral DNA by standard curve, plasmid standards (containing the viral MCP target; [41]) were used; the mean number of MCP copies from qPCR wells with amplification was used to express the viral load estimation.

Generalised, linear, mixed models (‘glmmTMB’ package; [42]) were used to test the effect of the sampling method on both the detection of *Ranavirus* infection (with a Binomial error distribution; logit link) and viral load (with zero-inflation and a ziGamma error distribution; log link). Individual fish were treated as a random factor, to account for the use of two samples from each fish. The relation between the viral load obtained with the two methods was tested with a Spearman’s rank correlation. Data were log-transformed for all viral load analyses, and here only positive individuals were used. All analyses were performed in R Studio, version 1.4.1717 [43].

## 3. Results

**Figure 2:**
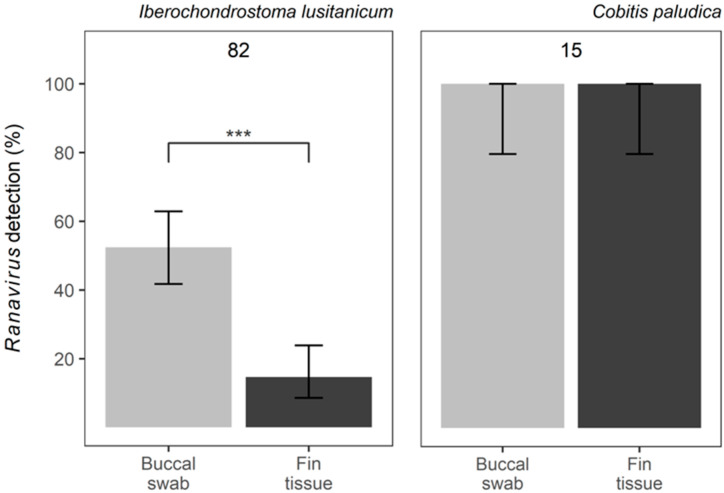
*Ranavirus* detection using two different methods: buccal swabs vs. fin tissue: proportion of samples resulting in positive detection of *Ranavirus* in *Iberochondrostoma lusitanicum* and *Cobitis paludica*. Top numbers reflect the total sample size. Error bars indicate the 95% confidence intervals and significance is denoted by *** *p* < 0.005.

A total of 82 individual fish of *I. lusitanicum* and 15 of *C. paludica* were sampled and tested. No mortalities were recorded. All individual fish of *C. paludica* tested positive for *Ranavirus* in both types of samples. For *I. lusitanicum*, the proportion of individual fish testing positive via a buccal swab or fin clipping was 52% (43/82) and 15% (12/82), respectively (Figure 2). This difference in prevalence was highly significant (*z* = −6.949, *SE* = 2.807, *p* = 3.67 × 10^−12^), with fin tissue being less effective in detecting *Ranavirus*. All individual fish that tested positive via fin tissue were also positive in the correspondent swab sample, while 31 of the 43 *Ranavirus* positives via swabbing did not amplify from fin clips.

**Figure 3:**
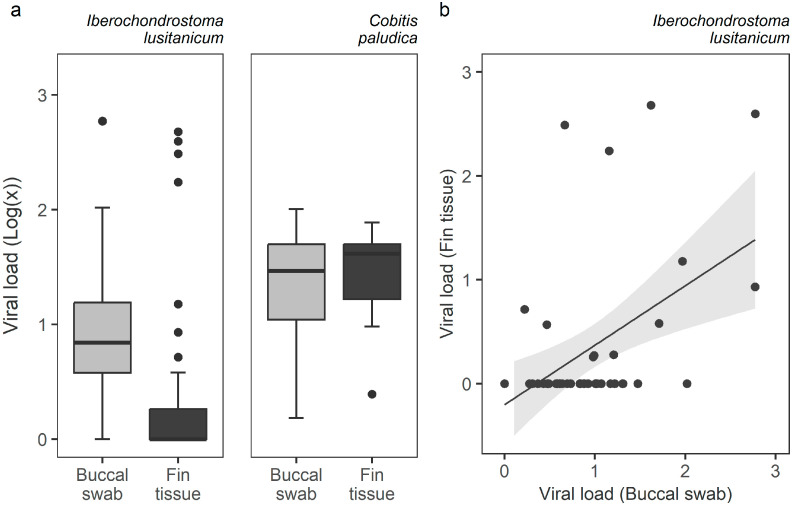
*Ranavirus* load obtained from buccal swab and fin tissue samples for *Iberochondrostoma lusitanicum* and *Cobitis paludica* individual fish that tested positive for *Ranavirus*: (**a**) boxplot of viral load by sampling method and species; (**b**) scatterplot showing a positive correlation between the viral load of buccal swab and fin tissue samples. Viral load is presented as a log (MCP copies per cell + 1).

The viral load in positive samples of *I. lusitanicum* ranged from 1.0–593.9 MPC copies (mean 22.0; standard deviation 79.1) in buccal swabs, and 1.8–477.8 (16.6; 54.5) in fin tissues. As for *C. paludica*, *Ranavirus* load ranged from 1.5–101.2 (9.4; 19.4) in buccal swabs, and from 2.5–77.1 (8.4; 15.2) in fin tissue. Thus, while the type of sample did not predict viral load in *C. paludica* (*t* = 1.024, *SE* = 0.091, *p* = 0.306), buccal swabs yielded a significantly higher viral load in *I. lusitanicum* (*t* = 4.862, *SE* = 0.383, *p* = 1.16 × 10^−6^) (Figure 3a). Nevertheless, the latter presented a positive correlation between the viral loads estimated from buccal swabs and fin tissue (*r_s_* = 0.36, *p* = 0.015) (Figure 3b). No correlation was found for *C. paludica* (*r_s_* = 0.28, *p* = 0.307).

## 4. Discussion

The results show that buccal swabbing, a minimally invasive technique, is a potential alternative for *Ranavirus* screening in freshwater fish. Both sampling methods detected *Ranavirus* DNA, suggesting an infection status in both fish hosts, although their efficacy varied between species. Whilst buccal swabbing and fin clipping performed similarly when detecting and quantifying *Ranavirus* in *C. paludica*, detection rates were greatly improved in *I. lusitanicum* buccal swabs compared to fin clips. Additionally, higher yields of viral DNA were retrieved from buccal swabs in *I. lusitanicum* (22.0 ± 79.1 compared to 16.6 ± 54.5 in fin tissue). These results suggest that contrary to fin tissue, the viral load in swabs can be obtained even at low values.

Although this study was not a controlled experiment, the findings point to ~70% false negatives when using fin clips to detect *Ranavirus* in infected *I. lusitanicum*. Swabbing the buccal cavity of a fish allows the swab tip to come into direct contact with the irrigated gills and epithelial cells, which the virus is known to target [44]. Meanwhile, the clip of the caudal fin is not as irrigated, consisting mostly of integument, mucous connective tissue and bone [45]. Moreover, because *Ranavirus* first replicates in the oral cavity [46], swabbing this area may result in increased chances of detecting viral particles in the early stages of infection. Ingestion, as a route of *Ranavirus* infection, has been broadly supported by other studies (eg. [47,48,49,50]). Skin, however, appears to become infected later as the infection progresses [46], which could also explain why fin clips resulted in lower detection. Therefore, we suggest that buccal swabbing may be a more appropriate and reliable technique to screen small-bodied freshwater fish for *Ranavirus*. It is worth noting that due to small sample sizes in *C. paludica*, we cannot rule out a failure to detect a difference in probability of detection between the two methods in this species [51].

The present findings go beyond previous studies, revealing that buccal swabbing is not only effective as a host DNA collection method [30], but can be used to detect and quantify *Ranavirus* with confidence in fish. The use of this technique has been validated for some amphibians, being just as, or even more, efficient in detecting *Ranavirus* strains than internal and external organs [33]. Yet, these results contrast with previous studies on herpetofauna [25,52]. However, while this difference could be attributed to the taxonomic assignment of the host species, in both studies the authors swabbed the oral cavity first, followed by the cloaca. Oral–cloacal swabbing may lead to the loss of viral particles, potentially acquired in the oral cavity, when rolling the swab inside the cloaca. Moreover, false negatives may be yielded by qPCR inhibitors associated with cloacal swab samples (similar to what has been reported by Das et al. [53]).

Overall, the buccal swabbing of fish for the detection of *Ranavirus* seems promising as a refined procedure, which depends neither on post-mortem samples, euthanasia of animals, nor invasive tissue removal by fin clipping. Given that our study was conducted in a natural system, using two threatened species, obtaining internal tissues becomes difficult and ethically questionable. However, further investigation using internal organs in a controlled experiment could corroborate the performance of this technique compared to lethal sampling. Nevertheless, our study demonstrates that buccal swabbing effectively detects *Ranavirus* even in asymptomatic fish. This is consistent with a recent study on clinically healthy frogs [33], suggesting the potential use of this methodology for detecting early stages of infections, although further work needs to be conducted in fish to confirm this hypothesis. This result becomes very relevant in field studies and when threatened species are involved, but also in the context of aquaculture; as a minimally invasive and consistent sampling approach, buccal swabbing could be ideal for long-term health surveillance, leading to a reduction in the harm and unnecessary culling of species of conservation concern.

## Data Availability

Any computer codes used to generate results reported in the manuscript, as well as raw data that support the findings of this study, are available on request from the corresponding author, without undue reservation.
